# Supplementary figures and images for: Zygote morphogenesis but not the establishment of cell polarity in Plasmodium berghei is controlled by the small GTPase, RAB11A
Source: PLoS Pathog. 2020 May 28;16(5):e1008091. doi: 10.1371/journal.ppat.1008091 (PMC7255598; doi:10.1371/journal.ppat.1008091)

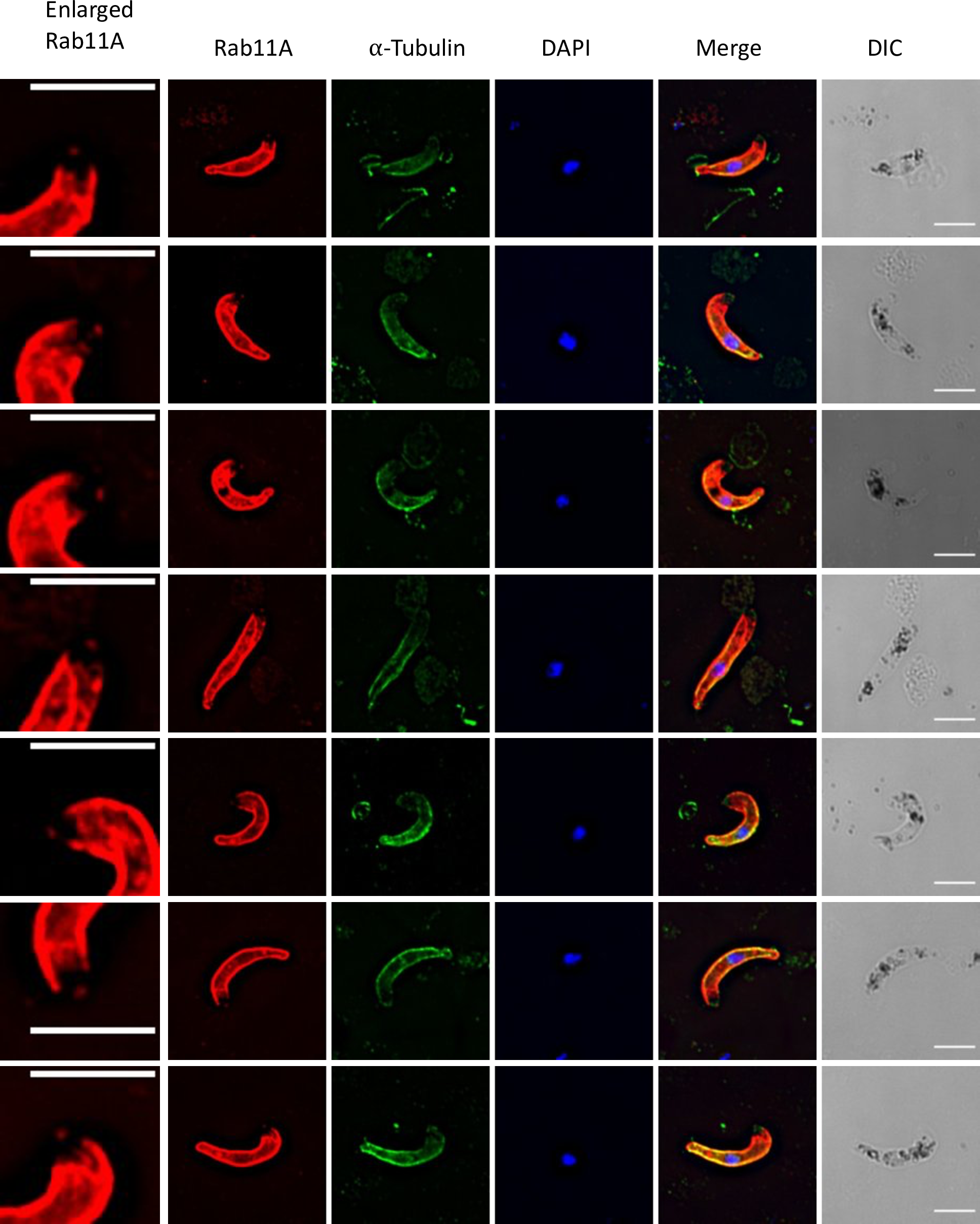

Supplement: S1 Fig — Localization of Rab11A in WT-GFPCON ookinete. The first column shows magnified images of apical tip of ookinete (taken from the images in the second column) stained with anti-PbRab11A antibody. Scale bar 5 μm. (TIF) [file ppat.1008091.s001.tif]

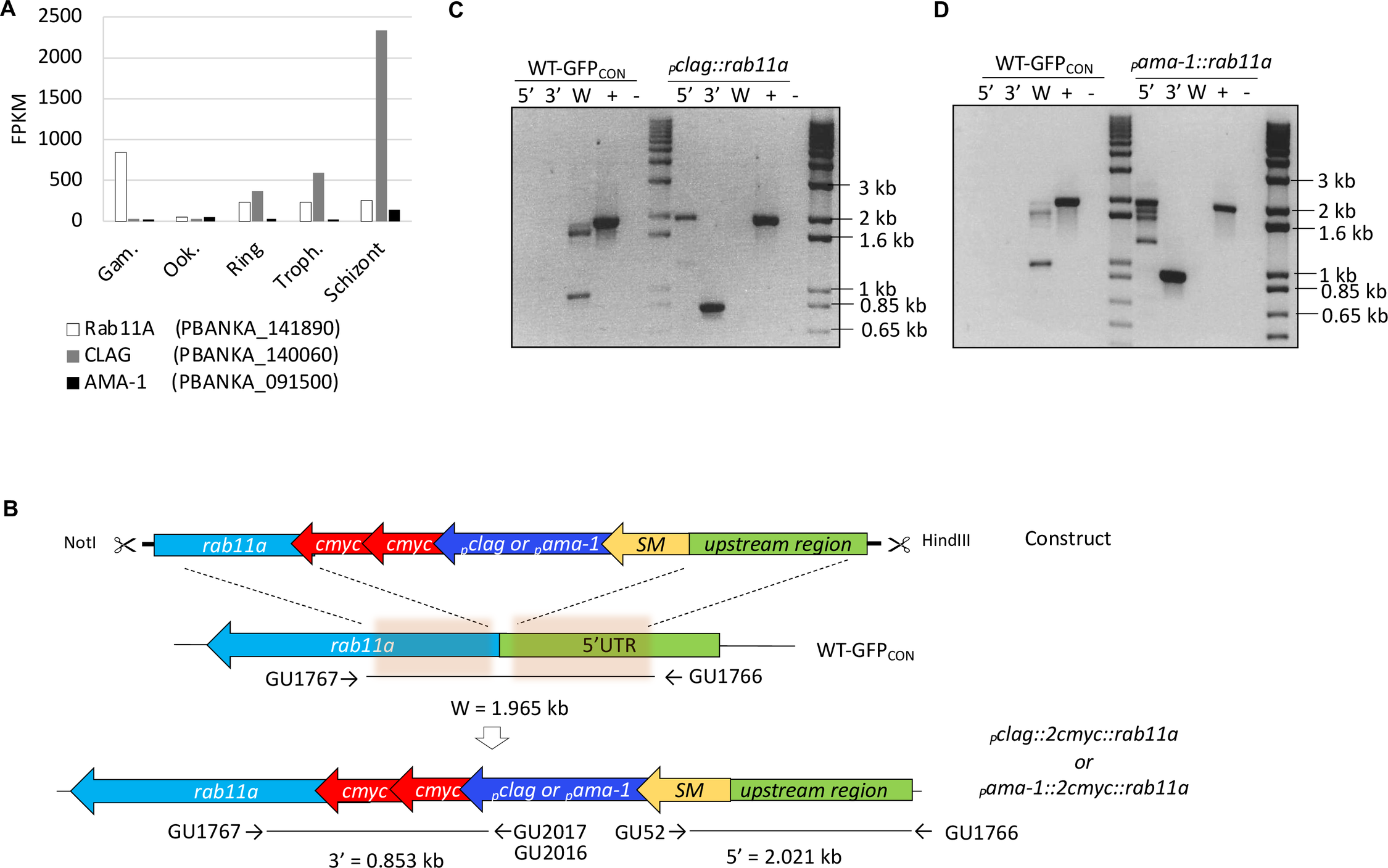

Supplement: S2 Fig — A. P. berghei RNA-Seq data for rab11a, clag and ama-1 shown in FPKM [49]. B. Schematic of the generation of pclag::rab11a and pama-1::rab11a parasites (Not to Scale). Diagnostic PCRs for integration of (C) pclag::rab11a and (D) pama-1:: rab11a constructs into WT-GFPCON gDNA, respectively, showing the 5’ and 3’ integration of respective constructs (PCR fragment as annotated in the schematic in B). W indicates a fragment present only in WT-GFPCON parasites. DNA ‘+’ is an unrelated positive PCR control (P28) and ‘-’ is a no DNA template negative PCR control. Abbreviations: USR, upstream region; SM, selectable marker. (TIF) [file ppat.1008091.s002.tif]

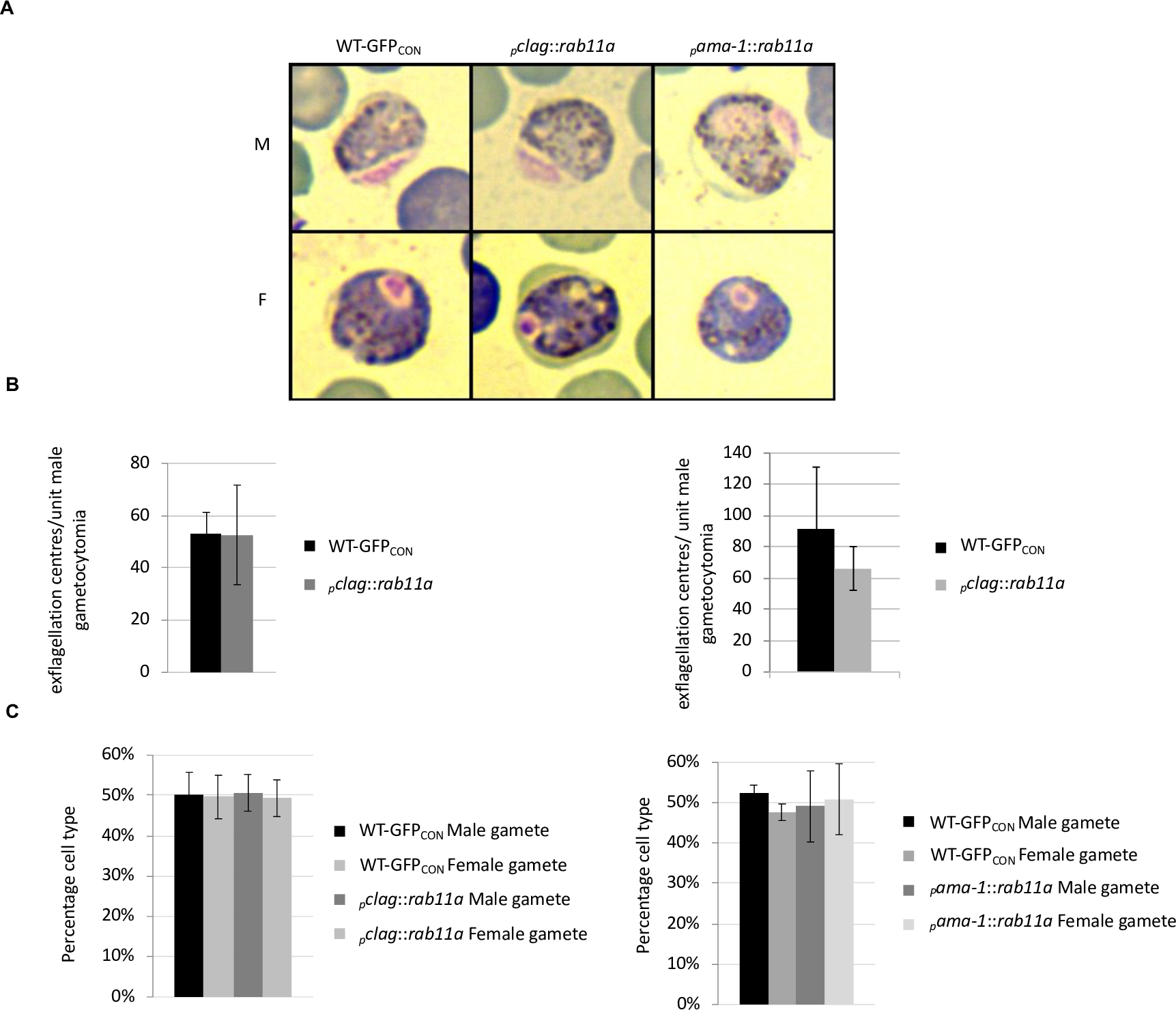

Supplement: S3 Fig — A. Images of Giemsa stained pclag::rab11a and pama-1::rab11a gametocytes; M, male gametocytes; F, female gametocytes. B. Exflagellation count of male gametocytes of pclag::rab11a (n = 4, mean +/-SD, two tailed student t test, p-value 0.9891) and pama-1::rab11a (n = 3, mean +/-SD, two tailed student t test, p-value 0.4337). C. Ratio of male to female gametocytes in pclag::rab11a (n = 6, mean +/-SD, two tailed student t test, p value 0.00046) and pama-1::rab11a (n = 3, mean +/-SD, two tailed student test, p-value 0.0111) parasites compared to WT-GFPCON. (TIF) [file ppat.1008091.s003.tif]

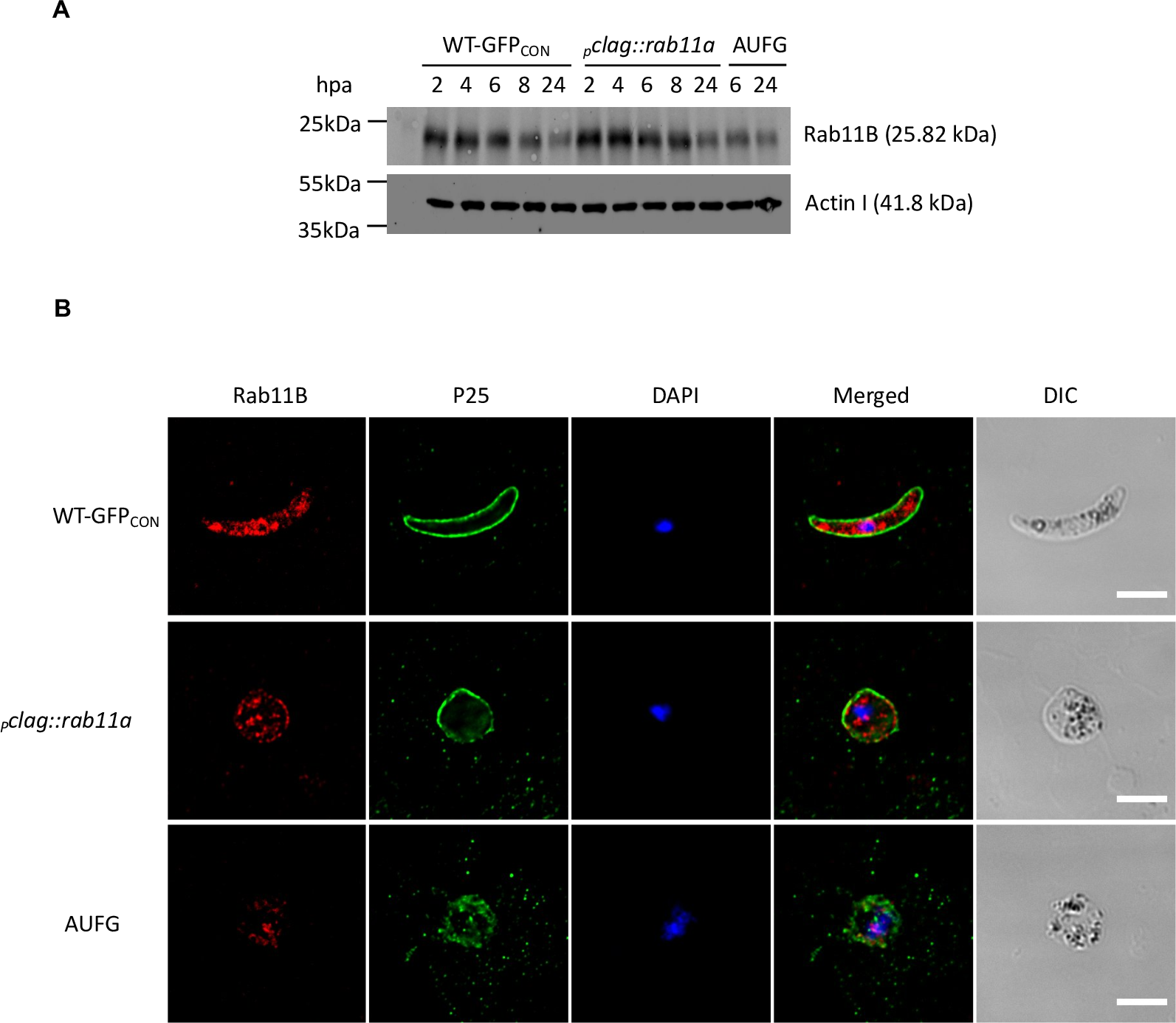

Supplement: S4 Fig — A. Western blot analysis of PbRAB11B expression following activation of gametocytes comparing wild type parasites with Pclag::rab11a and Activated Unfertilised Female Gametocytes (AUFG) across the time course of zygote to ookinete transition sampling at times indicated in hours. B. Immunofluorescence imaging of PbRAB11B expression at 24hpa in the same parasite lines when mature ookinetes should be present. Scale bar = 5μm. (TIF) [file ppat.1008091.s004.tif]

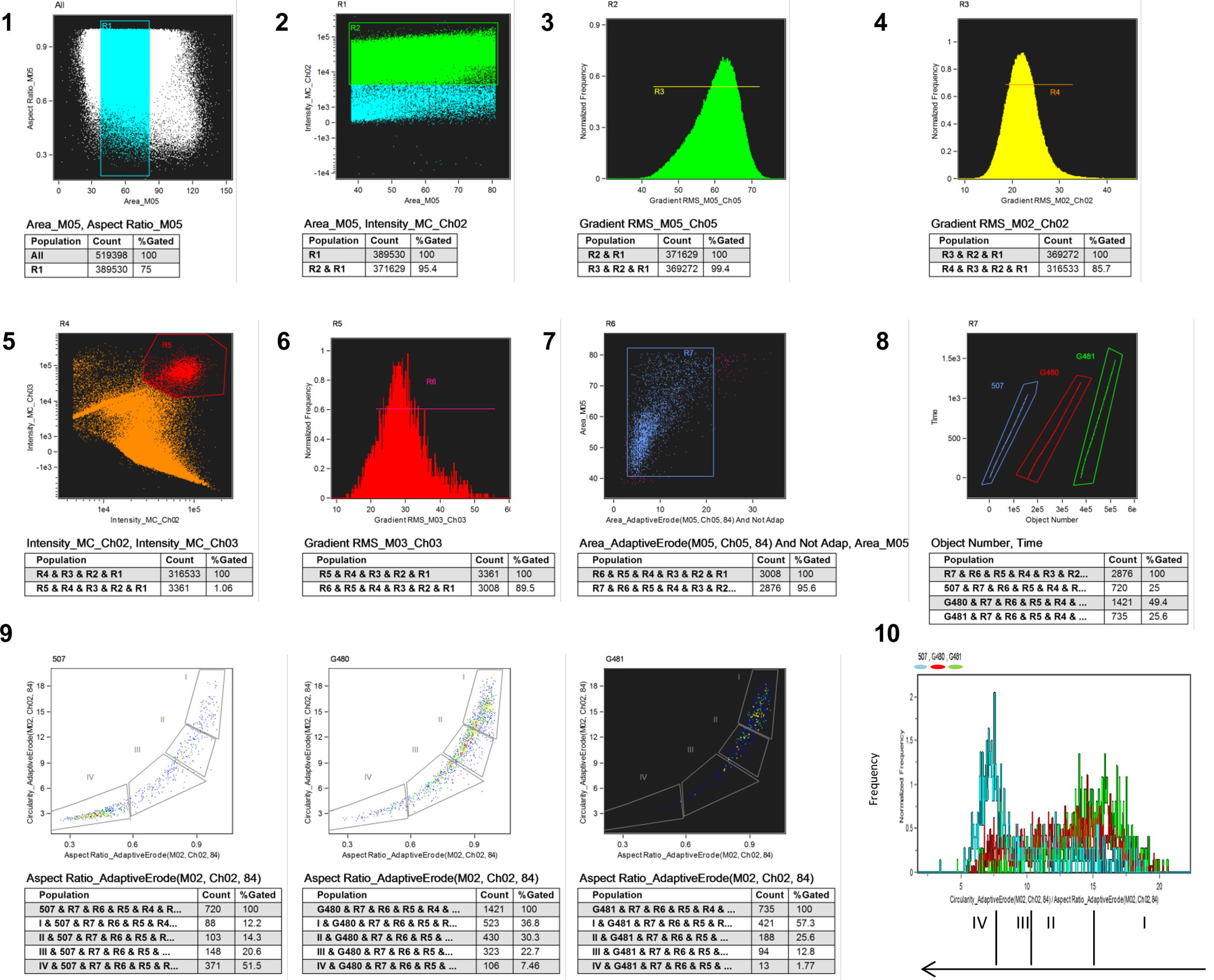

Supplement: S5 Fig — Dot plots and histograms showing the sequential gating strategy pipeline used to identify ookinetes and quantitate conversion rate. Files were acquired separately then merged in IDEAS software. Step 1 –gate R1 defined objects within a broad size range that includes infected RBC, gametocytes and ookinetes. Step 2- R2 includes cells positive for GFP. Step 3 –R3 gates on cells in focus on the Brightfield image. This gate is broad as we observe that some activated female gametocytes displayed a lower gradient RMS that is normal for focussed cells. Step 4—Gate R4 allows us to select cells in focus in the GFP channel. Step 5—Gate R5 selects for cells positive for P25-Cy3. The proportion of GFP positive cells that were also positive for P25-Cy3 was similar for the WT and the promoter swap lines. Step 6—The next gate selects cells also in focus in the P25-CY3 channel. GFP positive cells include asexual stages and non-activated gametocytes. Events positive for GFP and Cy3 not included for analysis include uninfected RBC that are autofluorescent as a result of the phenylhydrazine treatment given to enhance parasitaemia. Step 7—To help exclude images containing debris or overlapping cells a mask was generated to allow selection of cells where the area of the brightfield image was sufficiently larger than that of the area of the GFP image (Area_adaptiveErode(M05,Ch05) And not Area (M02,Ch02)). (Other strategies to exclude debris included using a spot count feature to identify images containing a single object, or the threshold feature to analyse only the object within the image that was within the size and intensity criteria. In some cases, images including doublets and debris were manually selected for exclusion from analysis). Step 8- To separate out the three merged files the object number vs time is plotted and three populations from the individual samples can be separated. Step 9 –Finally the circularity and aspect ratio features were generated on the adaptive erode (84%) [file ppat.1008091.s005.tif]

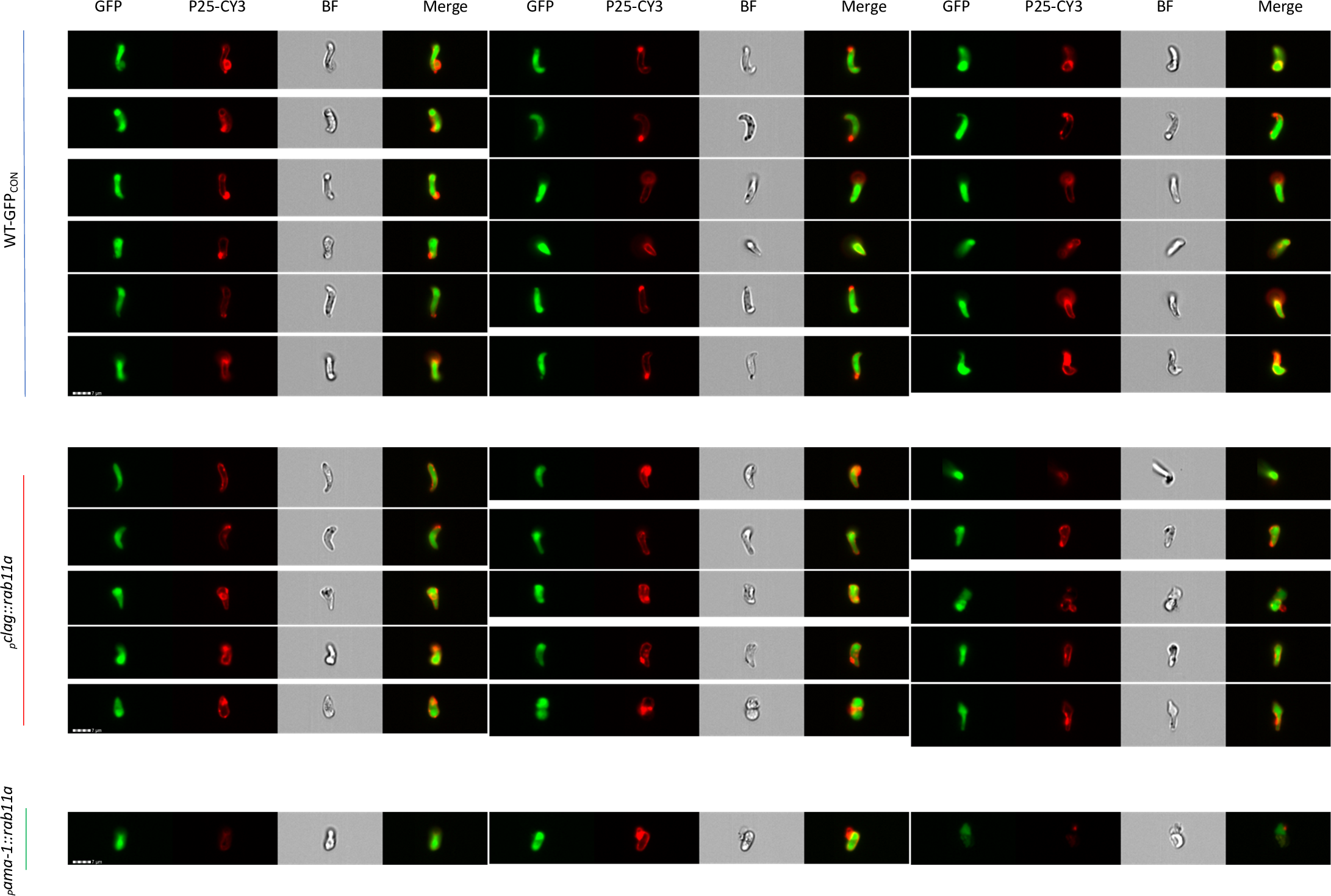

Supplement: S6 Fig — Gallery of representative images from the ookinete gate IV (Fig 2G) for the WT parasite line (top) and the promoter swap mutant lines. Scale bar (lower left) 7 μm. (TIF) [file ppat.1008091.s006.tif]

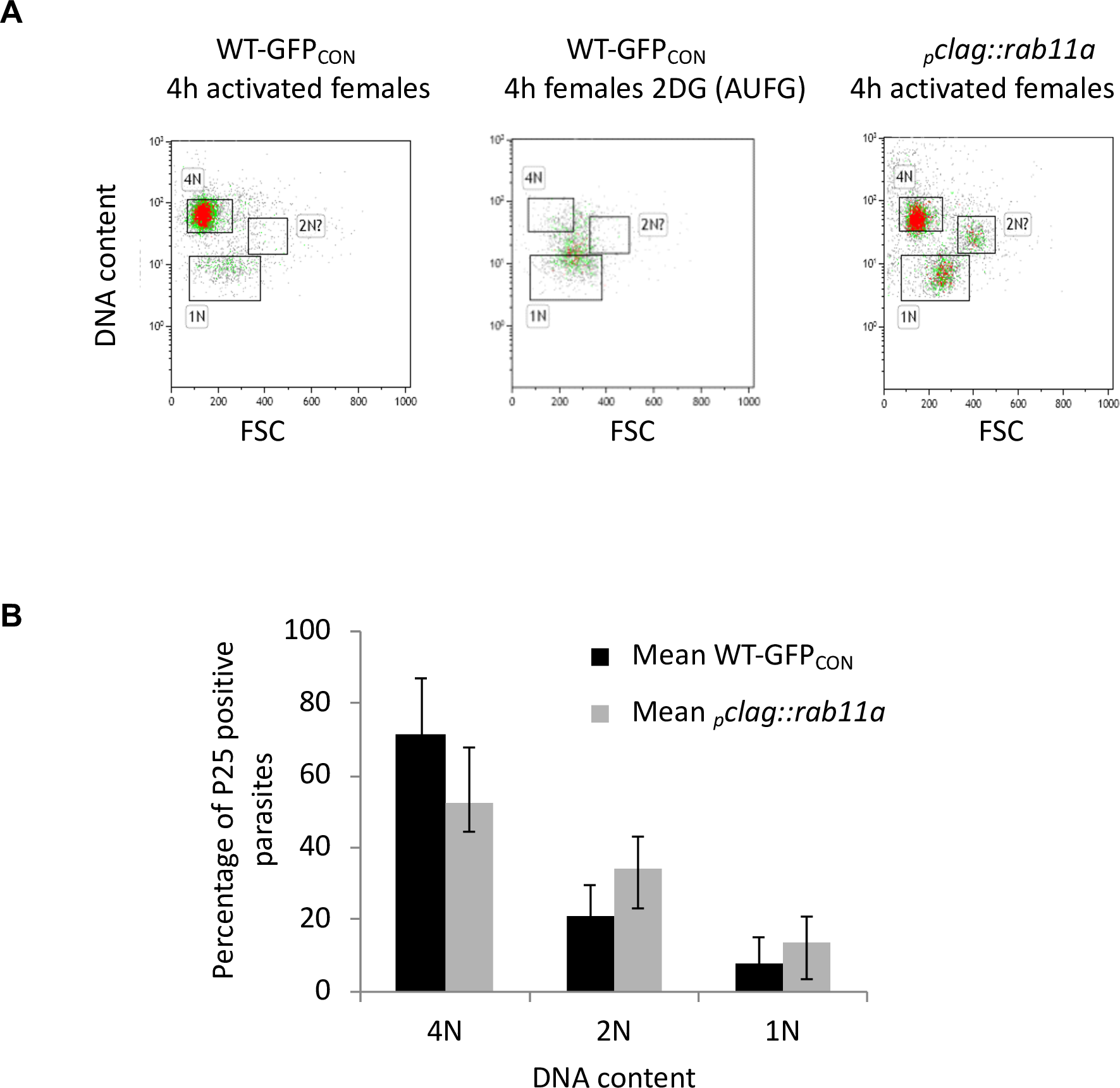

Supplement: S7 Fig — A. Flow cytometry analysis on FACsCYAN to illustrate DNA content of pclag::rab11a gametocytes 4hpa. Parasites stained with DNA stain Hoechst 33342 were gated for activation using anti-P25 antibody and DNA content in these compared to WT-GFPCON gametocytes 4hpa and WT-GFPCON activated unfertilized female gametes (AUFG) 4hpa. FACS plots showing results of one of three independent experiments. B. bar graph shows percentage of 4N (zygotes completed meiosis), 2N (fertilized female gametes, meiosis is incomplete or blocked) and 1N (gametocytes or asexual) parasites. Data from AUFG was used to verify the gating strategy (n = 3, mean +/-SD, two tailed student t test, p-value 0.129292). (TIF) [file ppat.1008091.s007.tif]

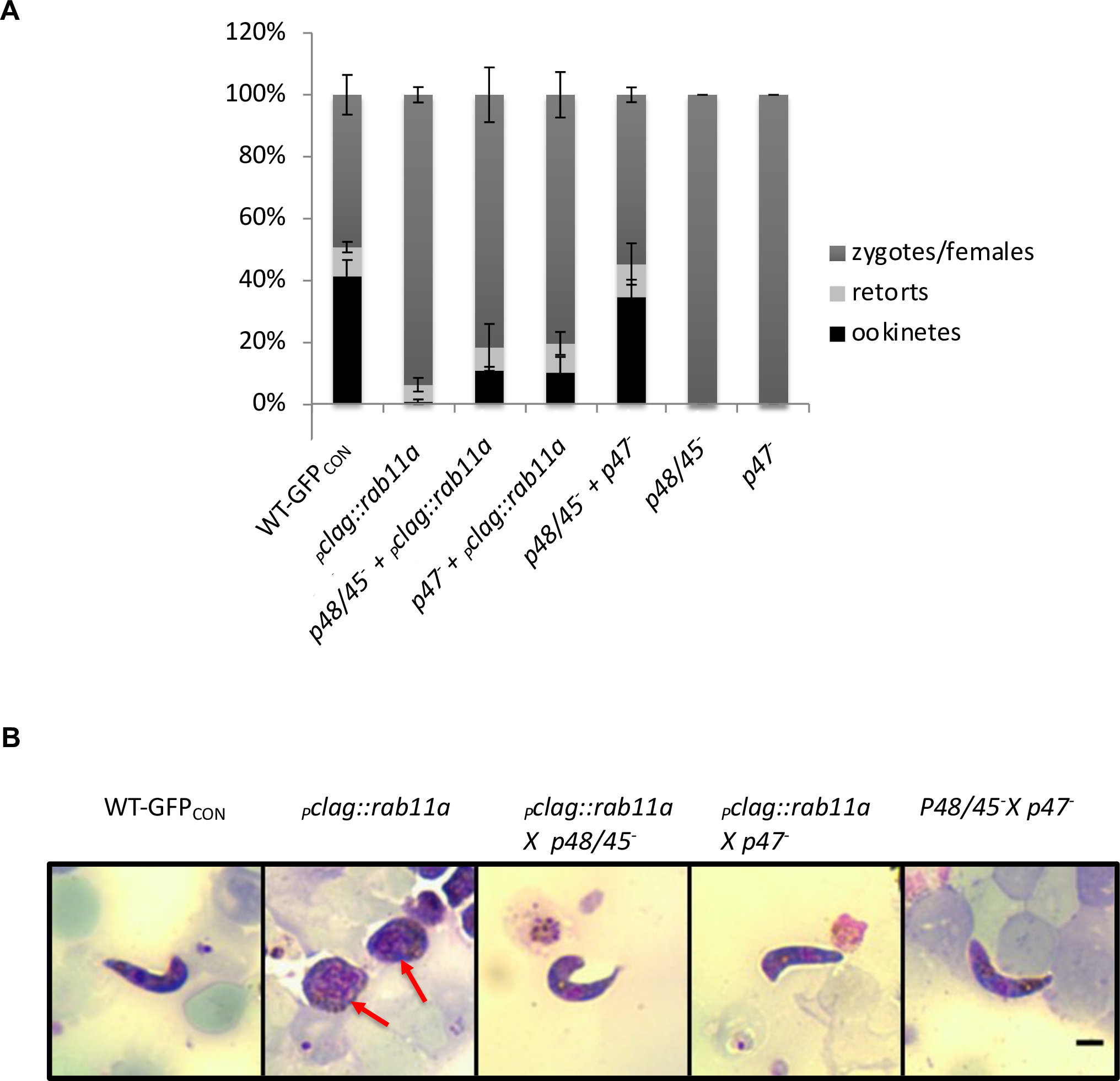

Supplement: S8 Fig — A. Cross-fertilization of pclag::rab11a with male defective (P48/45-) and female defective (p47-) mutants (n = 3, mean +/- SD, two tailed student t test, p value 0.0001) and B. Representative Giemsa images of ookinetes and zygotes obtained 24h after (cross) fertilisation. The decondensed nuclei of the pclag::rab11a zygotes are arrowed. Scale bar = 3μm. (TIF) [file ppat.1008091.s008.tif]

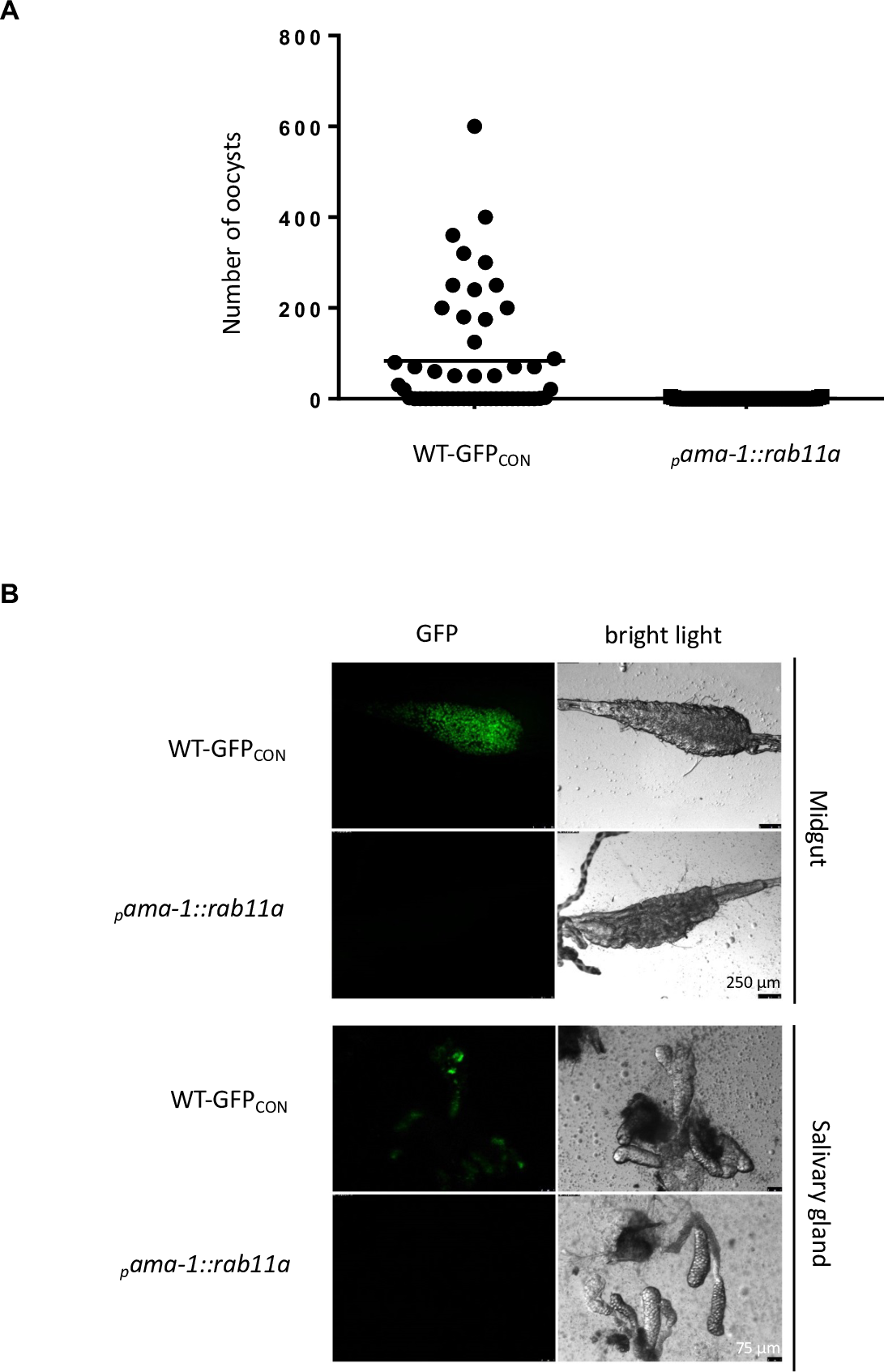

Supplement: S9 Fig — A. Plot of oocyst load in dissected midguts of WT-GFPCON and pama-1::rab11a fed mosquitoes (n = 2, two tailed student t test, p-value 0.0001). B. Fluorescent and bright field images of WT-GFPCON and pama-1::rab11a infected midguts and salivary glands. (TIF) [file ppat.1008091.s009.tif]

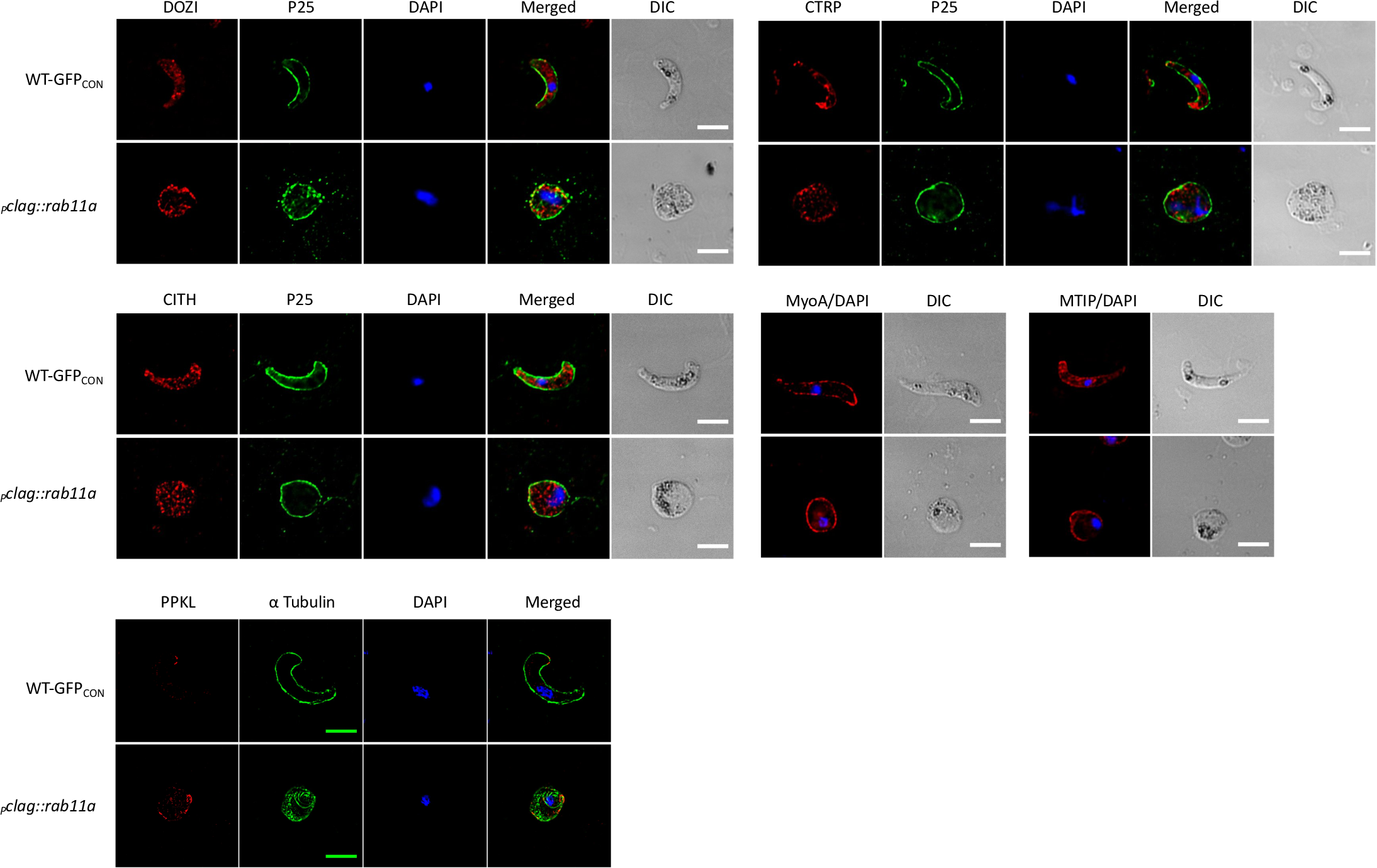

Supplement: S10 Fig — Fixed WT-GFPCON ookinetes and pclag::rab11a spherical ookinetes were probed with primary antibodies: anti-DOZI, anti-CITH, anti-PPKL, anti-CTRP, anti-MyoA and anti-MTIP antibodies mixed with either FITC-tagged anti-P25 or anti-α tubulin antibodies. Except PPKL, all images shown are single slice of Deltavision deconvoluted Z stack. For PPKL, single slice images of Z stacks obtained from ELYRA 3D SIM microscope is shown. Scale bar 5 = μm. (TIF) [file ppat.1008091.s010.tif]

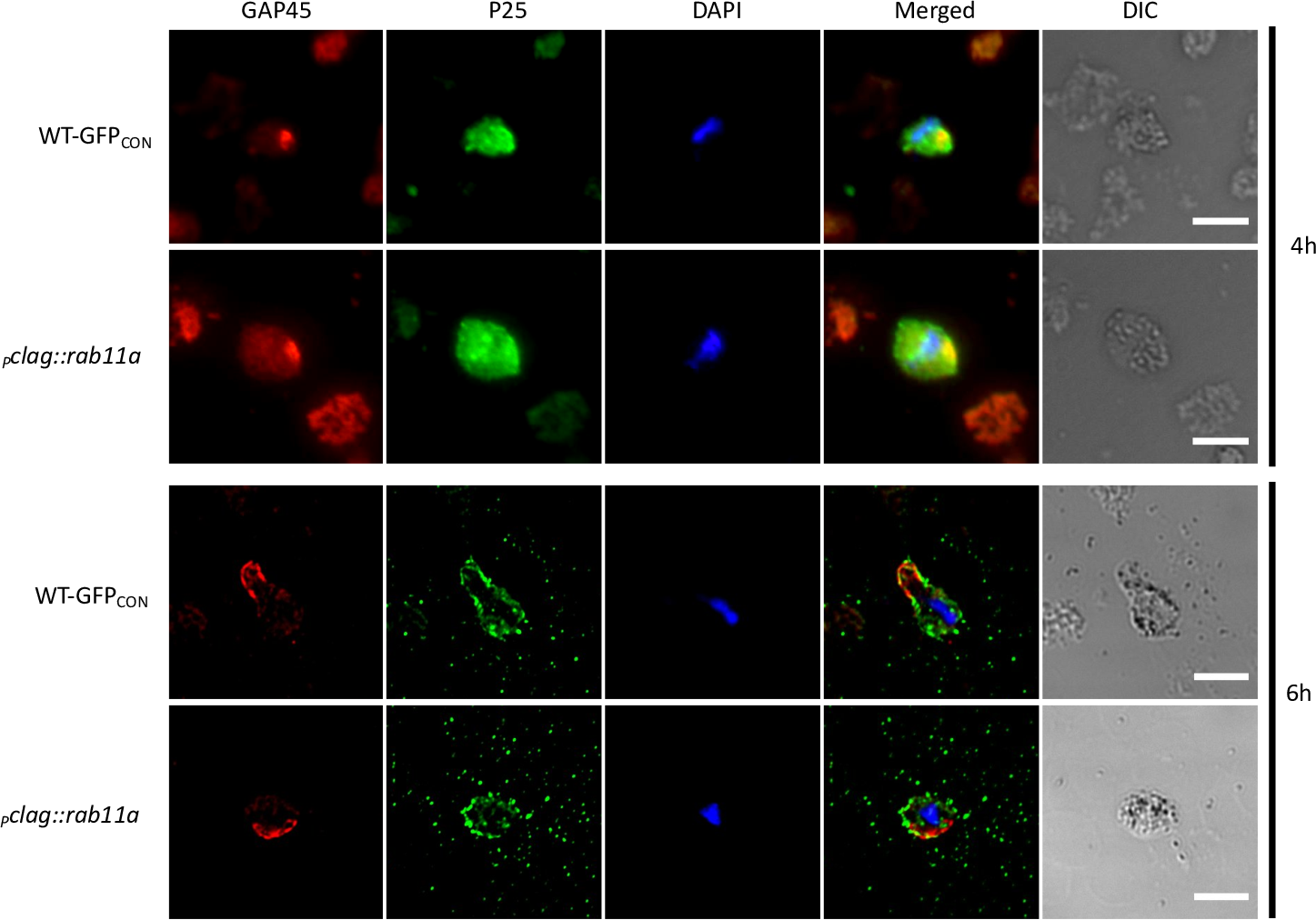

Supplement: S11 Fig — Time course immunofluorescence of WT-GFPCON and pclag::rab11a zygotes for GAP45 and P25 [4hpa timepoint images are taken through Axioplan and 6hpa timepoint images are single slices of deconvoluted Z stack taken from Deltavision microscope]. Scale Bar = 5 μm. (TIF) [file ppat.1008091.s011.tif]

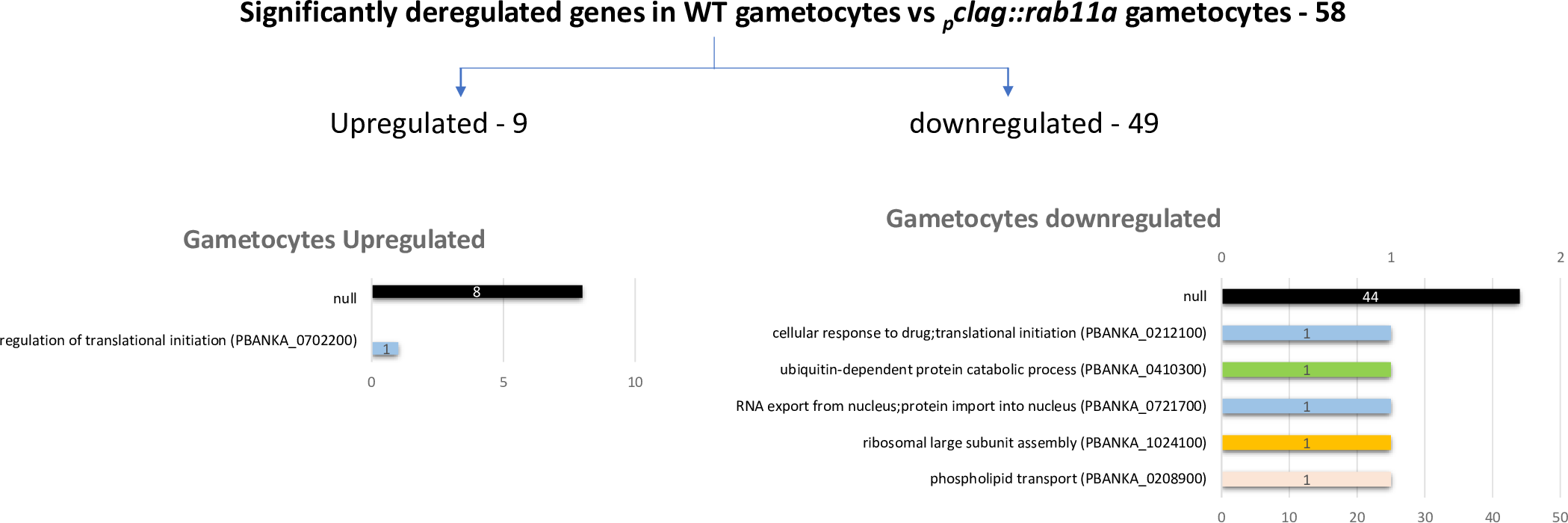

Supplement: S12 Fig — Summary of the expression levels of genes that are more than 2 fold altered in a comparison of wild type and rab11a KD gametocytes as assessed by RNAseq analysis. (TIF) [file ppat.1008091.s012.tif]

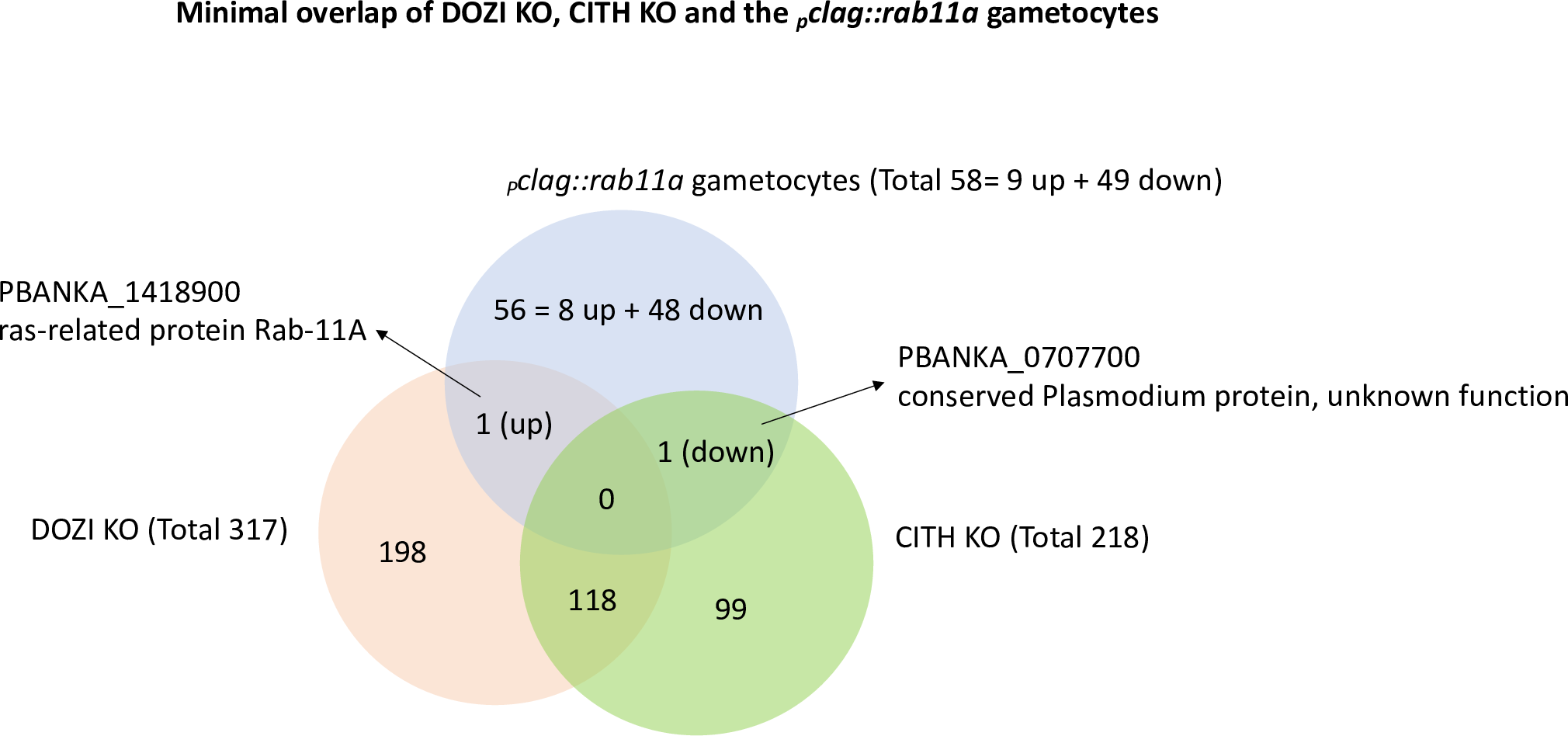

Supplement: S13 Fig — Venn diagram representation of the extent of similarity between the transcriptomes of pclag::rab11a, cithko and doziko gametocytes. (TIF) [file ppat.1008091.s013.tif]

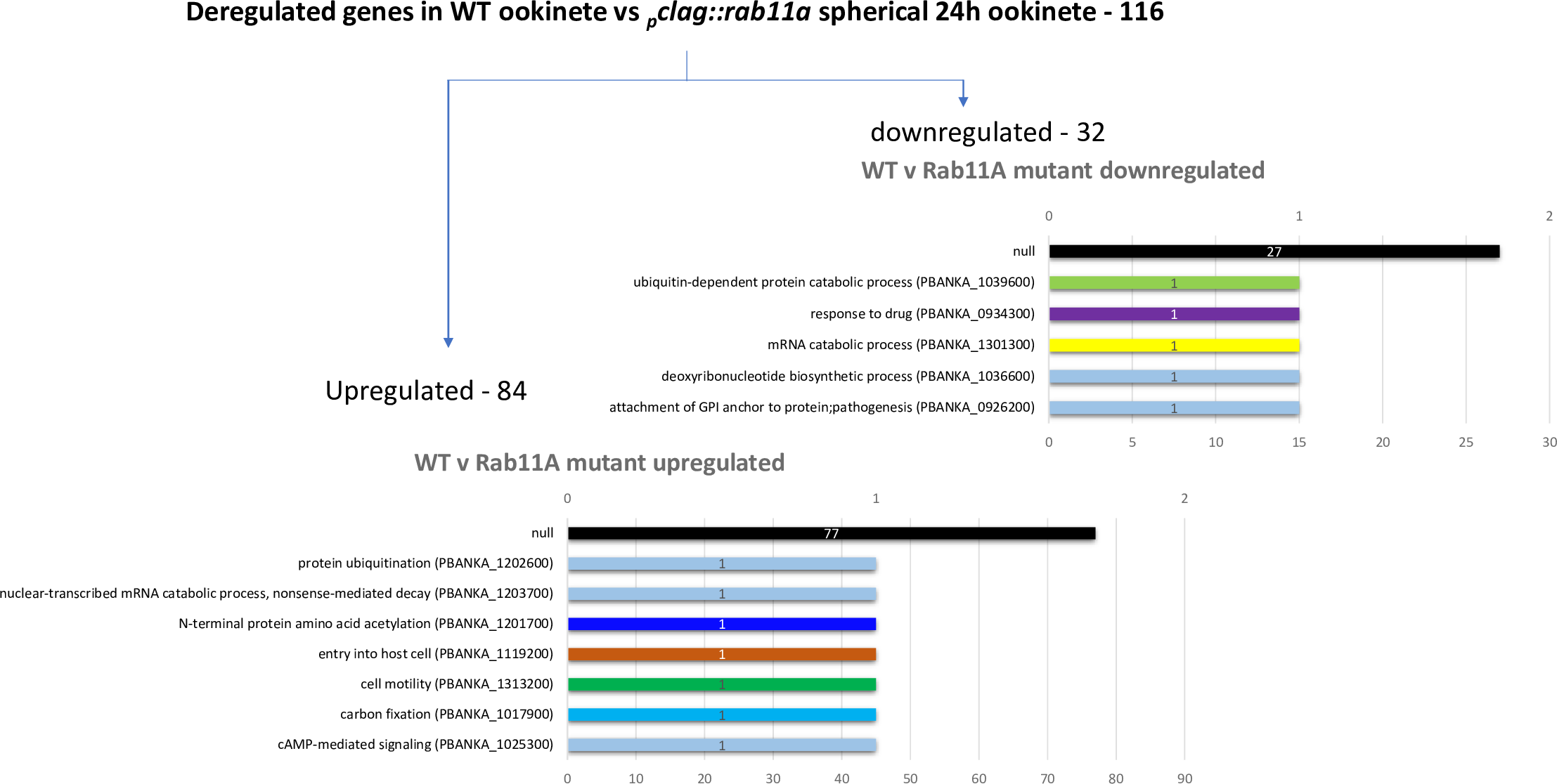

Supplement: S14 Fig — Summary of the expression levels of genes that are more than 2 fold altered in a comparison of wild type and rab11a KD ookinetes as assessed by RNAseq analysis. (TIF) [file ppat.1008091.s014.tif]
