# Supplementary material for: Zygote morphogenesis but not the establishment of cell polarity in Plasmodium berghei is controlled by the small GTPase, RAB11A
Source: PLoS Pathog. 2020 May 28;16(5):e1008091. doi: 10.1371/journal.ppat.1008091 (PMC7255598; doi:10.1371/journal.ppat.1008091)
Supplement: Attachment — Submitted filename: PPATHOGENS-D-19-01709 - [EMID-6a66051787e0d9f8] letter.doc [file ppat.1008091.s023.doc]

Glasgow, 18^th^ February, 2020,

**Zygote morphogenesis but not the establishment of cell polarity in *Plasmodium berghei* is controlled by the small GTPase, RAB11A**

H. Patil†, K.R. Hughes†, L. Lemgruber, N. Philip, N. Dickens, L. Starnes, A. P. Waters^*^

Dear Editor,

Please find associated with this letter the complete documentation that we have generated in response to the review process of the above-mentioned manuscript (your ref: PPATHOGENS-D-19-01709 - [EMID:6a66051787e0d9f8] Second revision).

Thank you for the opportunity to provide a more detailed explanation of the Chitinase secretion assay in Figure 6 of the manuscript. We have also included the data showing the apical staining of the RAB11A antibody on the *P. berghei* ookinete as requested creating a new Figure S1 and renumbering the rest of the Supplementary Figures.

We sincerely hope that the revised manuscript meets with the approval of all concerned and look forward to learning the outcome of this next step in the review process. We thank you for your time and attention to this.

Yours sincerely,
