# Supplementary material for: Zygote morphogenesis but not the establishment of cell polarity in Plasmodium berghei is controlled by the small GTPase, RAB11A
Source: PLoS Pathog. 2020 May 28;16(5):e1008091. doi: 10.1371/journal.ppat.1008091 (PMC7255598; doi:10.1371/journal.ppat.1008091)
Supplement: Attachment — Submitted filename: Authors response to Reviewer comments upload.docx [file ppat.1008091.s022.docx]

**Zygote morphogenesis but not the establishment of cell polarity in *Plasmodium berghei* is controlled by the small GTPase, RAB11A**

H. Patil, K.R. Hughes, L. Lemgruber, N. Philip, N. Dickens, L. Starnes, A. P. Waters

Reviewer's Responses to Questions with authors’ responses embedded

**Part I - Summary**
Please use this section to discuss strengths/weaknesses of study, novelty/significance, general execution and scholarship.


Reviewer #1: This manuscript describes a Plasmodium berghei knock-down of the gene encoding Rab11A. While this gene has previously been studied in Plasmodium the study offer new and unexpected insights into the function of the protein. The authors used a previously developed promoter swap approach to impair expression of the protein in the gametocyte stage and further onwards to the ookinete. This offered the possibility to study the function of the protein as a knock-out of the gene has been shown to be lethal. Two different promoters were used to generate the mutant parasites, with both showing the same phenotype. The mutants were analyzed in detail and the results show that they are blocked in the development from the round zygote to the extended ookinete. They show convincingly that polarity of the cell, defined as the presence of the apical complex, is not affected in the absence of the protein. The mutant parasites lack motility and secretion of micronemal proteins is also affected. The authors suggest that Rab11A is necessary for the trafficking of vesicles for the extension of the plasma membrane during ookinete development which is a reasonable hypothesis based on the data.
The study is of high quality and the conclusions are based on solid experimental evidence. Rab11A and its function in apicomplean parasites is of general interest. It is also of interest that I reviewed a study of the same protein in T. gondii and the publication of the two papers will provide a plethora of new data on this important protein in these parasites. I therefore recommend that the paper is accepted.
The authors also introduce a new method to study these cells using Imaging flow cytometry to obtain quantitative data.

Reviewer #2: The paper from Patil et al. Investigates the role of the small GTPase PbRab11A in Plasmodium berghei gametocytes and ookinetes using a promoter swap strategy to downregulate expression of PbRab11A in the targeted stages. Lack of PbRab11A had no effect on the production of gametes nor the fertility rate. Interestingly, lack of PbRab11A inhibited transmission of the parasite. This was correlated to a default in the zygote to ookinete transition, impaired motility and microneme secretion, and lack of morphological maturation.
This study provides valuable data regarding zygote and ookinete formation in Plasmodium berghei. It uses an elegant technological approach, i.e. promoter swap with promoters known to be silent in sexual stages. The subsequent experiments performed to investigate the consequences of knocking down Pbrab11A are well conducted and dissect all the crucial steps of the ookinete formation, with clear results.

Reviewer #3: The authors present an extensive characterisation of morphological developmental changes in ookinetes with Rab11A depletion. This builds on knowledge that the protein is unable to be knocked out in blood-stage parasites and uses molecular tools that enable propagation of a Rab11A positive cell line in blood stages that then losses Rab11A expression during the sextual stages. The major observations are that viable ookinete development is essential blocked, and cells fail to differentiate into the elongated ookinete form. This coincides with loss of motility, but interestingly much of the ultrastructure of the apical complex appears to be formed and intact. This is somewhat surprising given that elements of the IMC were presumed to rely on Rab11A for delivery and synthesis. Some evidence of a secretion defect is also presented, although these data are quite preliminary and undeveloped.

Overall, the study helps to define some elements of ookinete development that are not reliant on Rab11A (polarity establishment, apical complex assembly), and some that are (elongation and likely secretion competence for some cargo at least). A hypothesis is suggested that Rab11A is required for delivery of lipid for membrane expansion, and absence of this contributes to failure to elongate. This hypothesis is plausible but awaits testing. The data that suggests that secretion is affected by Rab11A depletion offers a further line of enquiry to resolve the roles of this protein, but this is represented by only one experiment, and the conclusions presented here are not well supported by the data at this stage (see below). This seems like an obvious and underexplored facet of Rab11A’s function in parasites, and perhaps one that is mechanistically linked with lipid delivery to the plasma membrane also.

The study has been executed very well, with some minor exceptions detailed below. It provides useful insight into elements of the process of cell formation, although ultimately a clear understanding of the role of Rab11A is outstanding. Nevertheless, this study will doubtlessly contribute to resolving this question. I would have liked to see a little more exploration of how the observations made in ookinete development might inform on the role and requirement of Rab11A in the blood-stage, and think that this would offer some further impact to this study.

**Part II – Major Issues: Key Experiments Required for Acceptance**
Please use this section to detail the key new experiments or modifications of existing experiments that should be absolutely required to validate study conclusions.

Generally, there should be no more than 3 such required experiments or major modifications for a "Major Revision" recommendation. If more than 3 experiments are necessary to validate the study conclusions, then you are encouraged to recommend "Reject".


Reviewer #1: None

Reviewer #2: no major issue

Reviewer #3: Some of the conclusions presented in the text were not well supported by the data.

A focus of Rab11A at the apical tip of ookinetes is described in the text but is not evident from Fig 1D. This looks like just the convergence of the periphery of the cell here.

We have discussed this comment at length as it is intriguing. This signal in ookinetes is in contrast to the absence of similar signal in merozoites where a similar convergence might be anticipated. We have examined our stock of images of ookinetes stained with the anti-RAB11A antibody and observe this signal in every image taken. We, therefore, believe that the signal is authentic. We have substituted a magnification of the particular image in the main figure for one that perhaps shows the signal more clearly at the scale of the original illustration and also inset a further magnification and arrow the signal to make sure that it is obvious to the reader. Lastly, we include here below a further selection of images that show the apical signal of RAB11A initially for the reviewers’ benefit alone but we would be happy to include those in the supplementary data if the reviewers would prefer.

The conclusion that a full and functional apical complex being developed in the mutant is going beyond what the data can report. Rather, by TEM the features of the apical complex can't be distinguished between the wildtype and mutants, leading to the hypothesis that it is full and functional. But at this point this hypothesis is untested.

We agree with the reviewer and have made what we hope will be an acceptable change in our wording on p8 :“ This suggested that an apparently complete apical complex was developed by *_p_clag::rab11a* zygotes consisting of the IMC, collar and internal organelles (see below) somewhat in contrast to the role for RAB11A in the delivery of surface and IMC markers in T. gondii [9]. The question of whether this complex is functional is not addressed in this study.

The quantitation of protein amounts shown in Westerns in Fig 6 is not compelling. Stated reductions in CTRP are not evident compared to DOZI. Only one experiment is shown. For this element of the study to be robust some further, independent data is required, and some numeric presentation would be ideal.

We completely agree that one experiment is not sufficient. It was our error that we did not describe this as a representative experiment. In order to completely convince the reviewers of the validity of this observation we have made more repetitions (3) and attempted to provide quantitation through imaging of the subsequent Western analyses. Furthermore, we have inadvertently misled the reviewers: detection of CTRP was performed using an antibody that detects the cytoplasmic C-terminus of the protein. Therefore, this reagent only detects full length, unprocessed CTRP and not the form that is shed into the culture supernatant. This serves as a further control of lysis of the parasite which is shown to be negligible. DOZI, as a very abundant cytoplasmic protein, serves as a very sensitive control for (sexual) parasite lysis which is again shown to be minimal. We reproduce the original western here but have made new samples and for simplicity run the assay omitting the CTRP detection which is now shown as a new Figure 6. The old image is pasted here for comparison.

The legend to the new Figure 6 has been changed accordingly omitting mention of CTRP.

**Part III – Minor Issues: Editorial and Data Presentation Modifications**
Please use this section for editorial suggestions as well as relatively minor modifications of existing data that would enhance clarity.


Reviewer #1: Page 4. The sentence “The role of RAB11A in apicomplexan parasites and Plasmodium falciparum rab11a (PF3D7_1320600) is essential…” needs revision

Agreed: changed to: “RAB11A has been shown to be essential in all apicomplexans tested including *Plasmodium* *falciparum rab11a* (PF3D7_1320600) [9]. RAB11A is expressed in all *Plasmodium* asexual blood stages with punctate protein localization in schizonts [7, 11, 13] that by analogy with *T. gondii* may be an association with rhoptries [14, 9]. “

Please note we have also changed the formatting of the references that were in separate brackets in two instances in that sentence.

Page 4. The protein MTIP is defined as Microtubule Interacting Protein while the correct definition is Myosin A Tail domain Interacting Protein.

Sorry! Corrected.

Page 5. “Rab11A controls the necessary step after biogenesis…” add a reference. Done. Agop-Nersesian et al 2009, PLoS Pathogens ref 9 in original - still ref 9 in revised text but cited at this point as well.

Page 7. “The nucleus of pclag::rab11a “ookinetes” appears decondensed”. This is not obvious in Fig. S7 where Giemsa stained cells are shown. Possibly Hoechst label would be more informative of this aspect. Either remove the sentence or show a better picture.

`We believe that this is actually shown nicely in Figure 2 by DAPI staining. Accordingly, we have directed the reader to these images as well in the original statement on p4.

“The nucleus of the *_p_clag::rab11a “*ookinetes” appears decondensed suggesting a qualitative impact on DNA replication (Figs 2A & S7).“

Page 8. The speed of WT ookinetes was measured and shown in Fig. 3C. In the text it says the average speed was 10 um/min while in the figure it is estimated at ~4 um/min.

We apologise for that confusion the actual figure should have been reported as 4 and has been corrected and the range of speeds (2-10 µm/min) also indicated in the text.

Page 11. “RAB11A is a highly conserved protein… host cell surface” it is not clear which host cell the authors refer to.

We have deleted “host” from the sentence - we were clearly carried away with host parasite interactions, yet this statement was intended to convey general activities of RAB11A

Page 12. “Spindle microtubules” are mentioned in several places referring to the microtubules of the ookinete. In my opinion this is not a correct term as spindle microtubules usually refer to the microtubules involved in mitosis and meiosis.

Agreed: we have deleted all instances of the term spindle in the text at this point and replaced it with the more accurate sub-pellicular.

Page 16. “Fertility of…” this sentence needs revision.

Revised – the sentence had residual text from an earlier edit for which we apologise. The revised sentence reads as follows: “Fertility of mutant gametes was assessed through *in-vitro* cross fertilization with *P. berghei* parasites able to produce either only fertile male (P47 KO) [39] or female (P48/45 KO) gametes [40], respectively.”

Fig. 1D. There is a non-labeled sporozoite for Rab11A in this Figure which shows protein expression during the life cycle by IFA. It is somewhat unexpected that the protein will not be expressed in sporozoites so there should be a comment in the text about this finding. The asterisk in the same picture is not defined.

Images in this section were stained with an anti-Rab11A antibody. This asterisked image was included as a negative control not exposed to antibody to convince that the staining was specific given the magnification is always difficult on sporozoites to generate clear images. It is confusing (especially between the legend does not refer to it). We have dropped the image from the manuscript it is more a hangover from the figure when it was compiled for a PhD thesis.

Figure 5F legend. Reverse the order of the sentence so that “upper” is first. Done

Fig. 5F. In the non-permeabilized samples P25 is found in a spot in the WT parasites, while the protein is found in the periphery of the whole cell in the mutant. In fact the latter pattern should reflect the normal distribution of P25 in the ookinete. Could this difference suggest that the protein is associated to the membrane more “loosely” in the WT ookinete? Please comment.

We believe that the P25 staining is around the periphery of the cells but it is certainly true that we often observe a concentration of stain at the basal end of the parasite when staining live cells. Overexposure of the images reveals the complete staining around the entire periphery but we avoided as much image manipulation as possible hence this appearance which can sometimes also be screen dependent. It was interesting that in the mutants this artefact was less apparent and so may indeed reflect some difference in the P25 “attachment” or perhaps indicate reduced membrane trafficking or fluidity.

Fig. S2C. The comparison of the ratios of male/female looks almost identical in the graph but the P values are very low. What exactly is compared here?

The p value (0.00046) for this experiment was actually very good – these data are taken from direct physical measurement of gametocytes in stained blood smears. We have included in the Materials and Methods section a description of the experimental procedure that was used to obtain these values on p17 in the section headed “***Plasmodium berghei* infections, cultures and stage enrichment**” as follows:

The male to female gametocyte ratio was calculated by examining under 100x oil immersion lens Giemsa stained slides made from tail blood taken from phenylhydrazine and sulfadiazine treated mice 72 hrs post-infection having similar parasitemia. Five different fields with well separated cells were counted from each of the two Giemsa slides per experiment per parasite line (i.e. ten fields per experiment per parasite line). Six biological replicates were performed for pclag::rab11a vs WT-GFP and four replicates for pama-1::rab11a vs WT-GFP.

The raw data for this are now shown in Table S1 and element C has been removed from Figure S2.

Supplementary video 1. It is very difficult to see the moving ookinete(s). As the video is from a field with many cells it would be preferable if an arrow indicates the moving ookinete. Or show at higher resolution just the cell of interest. In fact, the video does not add any information and could be removed.

We have removed this item

************************

Reviewer #2: 1/ The paper needs serious proofreading in order to ease its understanding, in particular (but not only) in the results section. Here are a couple of problems that need to be corrected:
- Fig 1D: why do you show a midgut sporozoite, which seems to not express PbRab11A (black)? If this is relevant, say it and discuss it.

Please see the answer to Reviewer 1 but you are correct – it was a negative control not exposed to anti- RAB11A antibody and has been removed to avoid this confusion.

- Fig. S1B: I believe, the general assumption for the abbreviation “UTR” is “UnTranslated Region”. The 5’ UTR thus corresponds to the region, which is comprised between the transcription start site and the ATG start codon of a gene, and thus excludes the promoter. The scheme as well as the text must thus be corrected so that there will not be any confusion possible on which part of the gene has been exchanged. What does mean SM?

The reviewer is correct. We have allowed the definition of UTR to slip to define a 2kb region upstream of the ORF which will of course include the strict definition of the UTR and usually sufficient promoter sequence to drive accurate expression of the gene. We have amended the legend and figures to reflect the comment using USR for UpStream Region. Thank you for the attention to detail, we were indeed sloppy. SM is selectable marker and is now given in the text of the legend and in the body of the main text on p 6 pf the revised manuscript where the description of the genetic modification occurs.

- Page 5, you mentioned in the results section that you chose the pclag::rab11A line for further analysis. However, the results of the pama-1::rab11A line are often included in the experiments (which does not hurt, of course), just modulate the sentence stating that you will only study the pclag::rab11A line.

Thanks and done. Now on p7 and the text reads: ”The *_p_clag::rab11a* line was arbitrarily chosen for the majority of the further analyses and western.....”

- Fig. 2D-E: mention at least that the anti-c-myc and anti-Rab11A detect a lower band in pclag::rab11A, not in pama-1::rab11A. How do you explain this difference?

We don’t have an explanation. It seems quite specific and may involve a processing event that we have not yet attempted to characterise. There are traces of a similar band in the *_p_ama-1::rab11A* line but it is certainly not as pronounced. We have added the following comment to the text on p7: “In addition to the full length protein, a slightly smaller RAB11A product is consistently detected by the anti-c-myc antibody in the *_p_clag::rab11a* line only although the reason for this is unclear but may represent a processing event that was not characterised further (Fig 2D).“

- Fig. S3, as I did understand it, refers to the expression of the PbRab11B paralog. The results section do not describe these results.

The text did mention these data and the relevant sentence is now highlighted on p7 of the track changes version of the revised manuscript.

- Fig. 2G: what about the stage IV? No expression of P25 (graph on the right).

These are parasites grouped according to shape and stage IV are the most mature achieving the authentic banana-like shape characteristic of this stage. As _p_ama1::rab11A parasites barely make the mature shape they fall outside this grouping so it is not no expression really it is more an absence of parasites in this sector of the analysis. For this experiment 9 events were included in this gate and manual inspection of the images suggested that they may be artefact (see images in right hand panel Fig 2I lowest panel). _P_ama1::rab11A is thus restricted to groups I-III and expresses P25. _p_clag::rab11A parasites do produce more parasites that can be classified by the machine as Group IV although inspection of the individuals in this section of (Fig2I) reveals them to still be far from idealised ookinetes but the mask in its current state of development does not discriminate between fully mature ookinetes and those arrested “proto-banana” shape mutants.

- Fig. 2I: I do not see it mentioned in the text.

Text added on p7 to cover this point: “The images of the parasites from the different groups confirmed the accuracy of the classifications (Fig 2I).”

- Fig. S6: although the pclag::rab11A line seems to be able to produce 4N parasites indeed, the parasites seem to produce less 4N cells (and conversely, more 2N and 1N cells). The sentence in the results section should be modulated.

We have modulated this statement as follows: The *_p_clag::rab11a* zygotes were able to complete meiosis (Fig S6) apparently with reduced success compared to WT parasites although this was not a statistically significant difference. The shaded area represents new text added on p7.

- Not being familiar with the proteins expressed by the sexual stages, I have a naïve question: you mentioned that the p47 KO line is female defective. I would thus expect it to produce only male gametocytes. However, I see a dark grey bar in the p47- column on Fig. S7A. I am confused.

Both the mating mutant lines p47ko and p48/45ko lines make mature gametocytes that can be activated to produce gametes but one or other gender of gamete is sterile therefore there will always be females present in the assay even if no fertilisation has taken place.

- Fig.3A and Fig. S8A: at which day were these oocysts numbered? What does represent the horizontal bar, the mean or the median? In the results, please refer to Fig. S8A, when appropriate.
Oocysts were counted on d11 and d14 as described in the M&M. Results for d14 are shown and the legend to Figure 3 now states this. Mean counts are indicated in all cases.

- Fig. 3D: how do you explain that so few WT-mCherry did move? Did you quantify these movements? We have removed the video from the manuscript on the recommendation of a different reviewer. The movie is on live unstained parasites. Some of the fluorescent events will therefore be unconverted WT females as we routinely have a conversion rate of only 50%-80%. Without a stain some asexual or unactivated parasites which also express mCherry may be visible. Some of the fluorescent cells may be autofluorescent RBC. Some ookinetes just don’t move within the time frame of the video.

- Fig. 4B: you should label the WT-GPF photos with IMC, Mt, M, etc, so that the comparison with the pclag::rab11A line is easier. Have done so where we can with confidence.

- Fig. 5A: GAP45, which is indicated to be 23.6 kDa (or Kd, but not kD) seems to migrate at 45-50 kDa… please check also Fig. 2D and Fig. 2E for the kDa. GAP45 is so named due to its apparent migration at 45kD despite its calculated Mw. All Mw indications in all figures changed to kDa (Figures, 1, 2, 5, 6)

- Fig. 5F: a few scale bars are missing. To make the figure clearer, you should also indicate on the figure that the upper panel corresponds to “no permeabilization” and the lower panel to “partial permeabilization”. The figure legend has been changed to indicate permeabilisation status and missing scale bars have been added. Imagestream scale bars (7 um) are included one on each gallery panel of images.

- Fig. 6: Quantification of the signals would allow to normalize on the DOZI signal resulting from lysis. Repeats of this experiment have been performed and an attempt at normalisation has been made and presented in a revised Figure 6. However, we would like to emphasise that we regard this as indicative rather than absolute. Please also see comments in response to Reviewer 1. Please add the “kDa” on the left. Done.

- The model should be included as Figure 7. The Golgi apparatus “G” is not indicated. We have removed “G = Golgi” from the figure as it is redundant. The apparatus is sketched in the left hand key and labelled and so doesn’t require further explanation. The model is now included as figure 7.

- Fig. S4: indicate the step numbers, as mentioned in the text for better clarity. The step numbers have been provided in the legend and a description added to the Materials & Methods section.

- Discussion, end of page 12: “SEM demonstrated a regular array of microtubule origins around the apical prominence.” Confusion with the TEM results? (as SEM cannot show internal structures). Sorry, both instances corrected!

- Discussion, end of page 13: if I understand well, you hypothesize that this is the lack of fusion of PbRab11A endosomes with the plasma membrane, which would be responsible for the lack of outgrowth of the apical bud, thus producing these roundish ookinetes. However, elongation of the cell would require a lot more lipids, i.e. its own lipid production, the endosome membranes are likely not sufficient to elongate the cell. Modification of both the lipid composition and the interactions with the cytoskeleton are also expected. This part of the discussion should be modulated. These are intriguing aspects that could be further investigated.

We appreciate the point and have attempted to incorporate the issues raised into a modified discussion at the relevant section at the bottom of p13 top of p14. There must be *de novo* synthesis of membrane as our data demonstrate that the ookinete is larger than the zygote. We hope the new text, reproduced below, covers this point adequately.

“In *_p_clag::rab11a* zygotes, all characterised developmental and structural markers are formed normally however, we hypothesise that PbRAB11A-mediated endosomes are responsible for delivering the additional plasma membrane required to form the ookinete as it is larger than the female gamete and early zygote. Normally such membrane structures would be both recycled and *de novo* synthesised and made available for the growing apical complex. In the absence of Rab11A this does not occur, neck markers are probably mis-localized however, apical bud formation is achieved but not further outgrowth.”

Question/suggestion out of the reviewing: would that be possible to develop super-resolution microscopy for this parasite? If yes, it would help a lot investigate the development of extracellular stages. We do intend to take the examination of this parasite line further into the SR area but feel that this is beyond the scope of the current manuscript.

2/ please perform a complete proofreading of the manuscript to 1) write the results in the past tense and 2) to correct the grammatical, orthography, punctuation problems (they are more numerous in the results section). Please see general response to the comment at the top of this document as this has been raised by a number of reviewers. We have attempted to resolve this issue as requested.

Reviewer #3: The results tend to be narrated in terms of what experiments/methods were used, and what data resulted, without a clear narrative of the questions that were driving the experiments. This made it a bit more cumbersome as a reader to decipher what was been tested and why. Please see general response to the comment at the top of this document as this has been raised by a number of reviewers. We have attempted to resolve this issue as requested.

Page 4 "during recycling endosomes" seems grammatically incomplete. Text changed to “the recycling of endosomes” to hopefully clarify/complete.

Fig 2F, I am not readily learning what the colours of the dots represent from this figure legend. Figure 2F is a density plot as commonly used to present flow cytometry data and a colour key has been provided.

Page 12: "Spindle microtubules" should be clearly defined to avoid confusion with mitotic spindles. Done

The grey versus white rendering of the cytoplasm in Fig 7 is a little unclear. We believe this must be a PDF file conversion issue as we’ve not attempted to introduce grey shading into the figure.
